# Convolutional Rebalancing Network for the Classification of Large Imbalanced Rice Pest and Disease Datasets in the Field

**DOI:** 10.3389/fpls.2021.671134

**Published:** 2021-07-05

**Authors:** Guofeng Yang, Guipeng Chen, Cong Li, Jiangfan Fu, Yang Guo, Hua Liang

**Affiliations:** ^1^Institute of Agricultural Economics and Information, Jiangxi Academy of Agricultural Sciences, Nanchang, China; ^2^Jiangxi Engineering Research Center for Information Technology in Agriculture, Nanchang, China

**Keywords:** imbalanced dataset, convolutional neural network, image classification, feature fusion, rice pests and diseases

## Abstract

The accurate classification of crop pests and diseases is essential for their prevention and control. However, datasets of pest and disease images collected in the field usually exhibit long-tailed distributions with heavy category imbalance, posing great challenges for a deep recognition and classification model. This paper proposes a novel convolutional rebalancing network to classify rice pests and diseases from image datasets collected in the field. To improve the classification performance, the proposed network includes a convolutional rebalancing module, an image augmentation module, and a feature fusion module. In the convolutional rebalancing module, instance-balanced sampling is used to extract features of the images in the rice pest and disease dataset, while reversed sampling is used to improve feature extraction of the categories with fewer images in the dataset. Building on the convolutional rebalancing module, we design an image augmentation module to augment the training data effectively. To further enhance the classification performance, a feature fusion module fuses the image features learned by the convolutional rebalancing module and ensures that the feature extraction of the imbalanced dataset is more comprehensive. Extensive experiments in the large-scale imbalanced dataset of rice pests and diseases (18,391 images), publicly available plant image datasets (Flavia, Swedish Leaf, and UCI Leaf) and pest image datasets (SMALL and IP102) verify the robustness of the proposed network, and the results demonstrate its superior performance over state-of-the-art methods, with an accuracy of 97.58% on rice pest and disease image dataset. We conclude that the proposed network can provide an important tool for the intelligent control of rice pests and diseases in the field.

## Introduction

In modern agricultural production, the accurate classification of crop pests and diseases is essential for their prevention and control. China is the largest rice producer and consumer in the world, accounting for one-third of the global total. Rice is the staple food of more than 65% of the Chinese people (Deng et al., 2019). However, pests and diseases always accompany the process of rice planting and production (Laha et al., 2017; Castilla et al., 2021). The prevention and control of rice pests and diseases could be greatly improved through their accurate classification.

**Figure 1:**
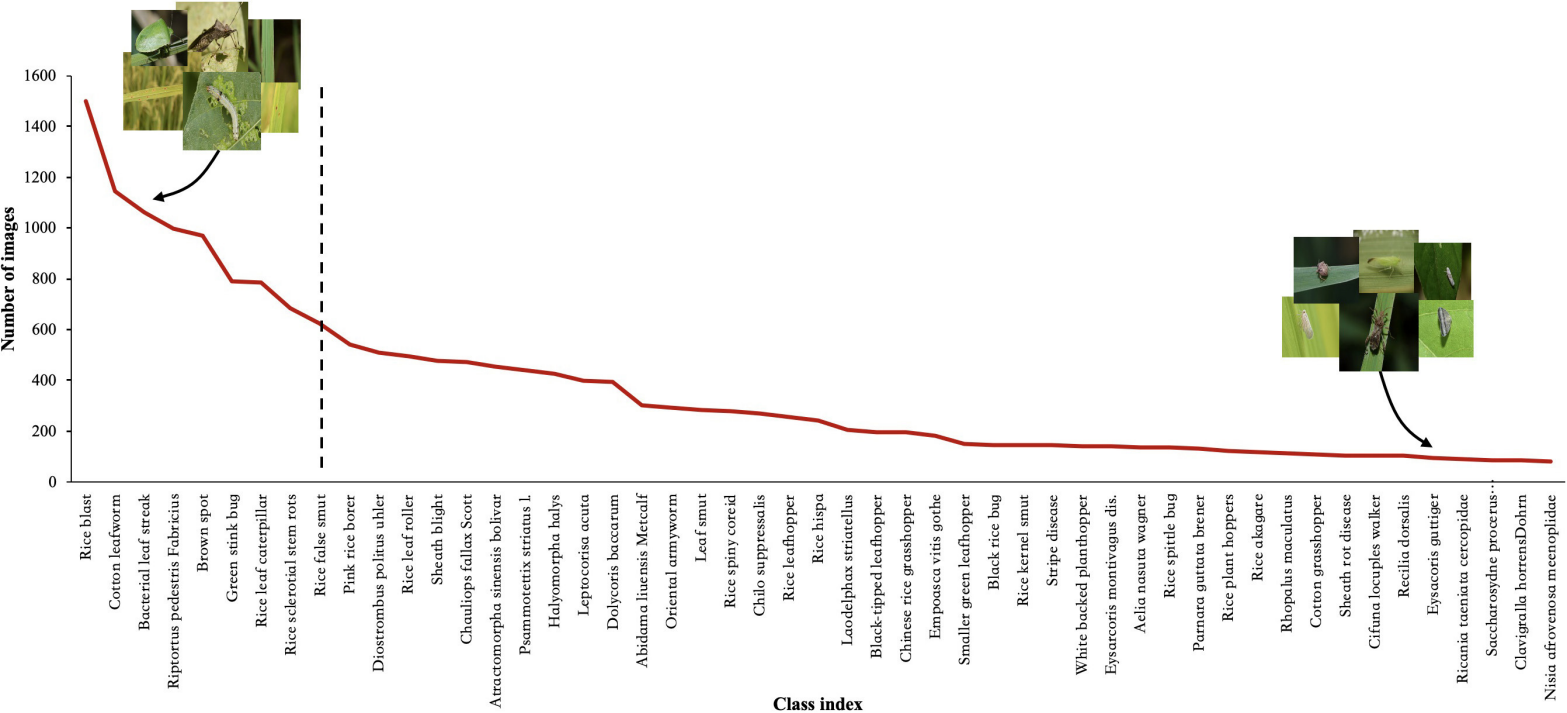
The imbalanced phenomenon of rice pest and disease images collected in the field.

Research on deep learning (DL) technology to classify crop pest and disease images has been emerging in recent years, and the relevant experimental results have demonstrated its success in performing classification (Li et al., 2020; Wang et al., 2020; Yang et al., 2020b). However, there is no doubt that these experimental results are inseparable from the high-quality datasets used or constructed by the researchers. We know that the size of the dataset can have a significant impact on the accuracy level of the image classification, because only the use of large-scale datasets can improve the accuracy of any DL model (Hasan et al., 2020). Most previous studies use small-scale, roughly balanced rice pest and disease image datasets created under laboratory conditions (Bhattacharya et al., 2020; Burhan et al., 2020; Chen et al., 2020, 2021; Kiratiratanapruk et al., 2020; Mathulaprangsan et al., 2020; Rahman et al., 2020). These datasets are used to emphasize or reveal the efficiency of the proposed method for diagnosing rice diseases and pests. Because these datasets contain several rice pest and disease categories and a small number of images per category, so the effect on classification is often a better performance. Compared with these image classification datasets, however, the distribution of real-world datasets is usually imbalanced and long-tailed. The number of images varies greatly between categories, and most image categories occupy only a small part of the dataset, such as ImageNet-LT (Liu et al., 2019), Places-LT (Samuel et al., 2021), and iNaturalist (Horn et al., 2018). Since rice pest and disease images collected in the field are affected by many practical factors, such as the incidence of pests and diseases, the region of occurrence, and so on, these factors often lead to an imbalanced distribution of the dataset, as shown in Figure 1. When using this dataset, DL methods cannot achieve high classification accuracy due to the problem of imbalanced distribution.

Most of the researches on DL for rice pest and disease classification uses a convolutional neural network (CNN) based on transfer learning technology (Burhan et al., 2020; Chen et al., 2020, 2021; Mathulaprangsan et al., 2020). Although these models have achieved a high level of accuracy in their respective studies, they rely mainly on two dataset features to achieve their results. First, the limited size of the dataset: the number of images ranges from dozens to hundreds, and image labeling usually requires professional knowledge and much annotation time. Second, there may be large or small differences in the number of images for different categories in the dataset. If these models are applied to real-world datasets, two challenges will inevitably be encountered. First, simple CNN models have difficulties learning the distinguishing features of different rice pests and diseases, and are insensitive to the discriminative regions in the image. It is difficult to locate the various organ parts of the pest object, and the small difference between different diseases will also affect identification of the location distribution. Second, due to the imbalance of different categories in the dataset, it is difficult to achieve a high level of classification accuracy for all rice pests and diseases, using only simple CNN models.

An effective method of solving the problem of dataset imbalance is a category-rebalancing strategy, which aims to alleviate the imbalance of the training data. In general, category rebalancing strategies can be divided into two groups: re-sampling (Lee et al., 2016; Shen et al., 2016; Buda et al., 2018; Pouyanfar et al., 2018) and re-weighting (Huang et al., 2016, 2020; Wang et al., 2017; Cao et al., 2019; Cui et al., 2019). Although rebalancing strategies have been shown to improve accuracy, they have side effects that cannot be ignored. For instance, such methods can, to some extent, impair the ability to represent DL features. Specifically, when the data imbalance is very serious, re-sampling has the risk of over-fitting the tail data (over-sampling) and under-fitting the entire data distribution (under-sampling). As for re-weighting, the original distribution is distorted by directly changing or even reversing the data presentation, which can damage feature representation. Experiments have shown that only the classifier should be rebalanced to rebalance an imbalanced dataset (Kang et al., 2020; Zhou et al., 2020). The distribution of the original categories in the dataset should not be used to change the distribution of image features or the distribution of category labels during feature learning because they are essentially uncoupled.

In order to improve the performance of rice pest and disease classification, we propose a convolutional rebalancing network (CRN), which includes a convolutional rebalancing module (CRM), an image augmentation module (IAM), and a feature fusion module (FFM). In the CRM, a uniform sample is used to extract the features of the images in the dataset, while a reversed sample is used to improve feature extraction of the categories with fewer images in the dataset. Based on these two modules, the IAM is designed to augment the training data effectively. To further enhance the performance of rice pest and disease classification, we also design the FFM, which fuses the image features learned by the CRM and ensures that the feature extraction of the imbalanced dataset is more comprehensive.

We evaluate the proposed network on the newly established large-scale dataset collected in the field, the rice pest and disease image dataset (RPDID), which contains 18,391 wild rice pests and disease images in 51 categories. Experimental results show that our network has a better classification performance than other competing networks on RPDID. In addition, a large number of verification experiments and ablation studies demonstrate the effectiveness of customized designs for solving imbalance problems in the distribution of rice pests and disease images.

The main contributions of this work are the following:

1. Based on the combination of the two sampling methods, we propose a novel convolutional rebalancing module for comprehensively extracting the features of the large-scale imbalanced dataset of rice pests and diseases to exhaustively boosting classification.
2. We design an image augmentation module, which mainly generates attention maps to represent the spatial distribution of discriminative regions, and extracts local features to improve the classification effect. Based on attention maps, we propose two methods of a region crop and a region cover to augment the training data effectively. Correspondingly, a feature fusion module is developed for adjusting feature learning and classifier learning, combined with the training of our network.
3. Experiments in the large-scale imbalanced dataset of rice pests and diseases and five related benchmark visual classification datasets demonstrate our proposed network can significantly improve the classification accuracy of imbalanced image datasets, which surpasses previous competing approaches.

## Related Work

In this section, we review related work on image classification of rice pests and diseases, imbalanced datasets, and image augmentation.

### Image Classification of Rice Pests and Diseases

The classification of rice pests and diseases has always been a hot topic for researchers, and many methods have been designed to identify different pests and diseases. In recent years, researchers have tended to use convolutional neural networks to solve the problem of identification and classification.

Most of this research has been concerned with only a few rice disease or pest categories (Bhattacharya et al., 2020; Chen et al., 2020, 2021; Kiratiratanapruk et al., 2020; Mathulaprangsan et al., 2020). Only Rahman et al. (2020) studied simultaneously five categories of rice diseases and three categories of rice pests, but these are far from covering common rice pest and disease categories. In addition, it should be noted that the datasets used in these studies are small, generally hundreds to no more than a thousand. Moreover, experimental results show that these methods can only achieve an ordinary classification performance. This is because, without a special network design, it is difficult for them to overcome the impact of an imbalanced dataset on the classification results and the difficulty of locating discriminative regions. We conclude that experiments based on small-scale datasets always achieve ordinary classification results, and, also, that the generalization of the model is often poor.

Among the methods used to identify and classify rice pests and diseases, there are traditional multilayer convolutional neural networks (Lu et al., 2017) and the fine-tuning methods of VGG-16, Inception-V3, DenseNet, and so on, based on transfer learning (Burhan et al., 2020; Chen et al., 2020, 2021; Mathulaprangsan et al., 2020). There is also the direct use of the popular object detection algorithms Faster R-CNN, RetinaNet, YOLOv3, and Mask RCNN, either to experiment with rice pests and diseases or to optimize these algorithms before performing experiments. However, these object detection algorithms depend on the location of parts or related annotations (Kiratiratanapruk et al., 2020). A two-stage strategy has recently been developed to perform a more refined classification of rice pests and diseases (Bhattacharya et al., 2020; Rahman et al., 2020). However, the classification performance of these methods is mostly average, because, without a special design, it is difficult for these methods to locate discriminative regions and to classify pest categories accurately. It is noteworthy that these studies did not investigate whether the balance of the dataset had an impact on the classification results.

### Imbalanced Datasets

The most effective method of solving the problem of dataset imbalance is the category rebalancing strategy. As one of the most important category rebalancing strategies, the resampling method is used to achieve a sample balance on the training set. The resampling method can be divided into oversampling of few samples (Shen et al., 2016; Pouyanfar et al., 2018) and undersampling of multiple samples (Lee et al., 2016; Buda et al., 2018). However, oversampling can overfit a category containing a small number of images (a minor category) and cannot easily learn more robust generalization features; therefore, it often performs worse on a seriously imbalanced dataset. On the other hand, undersampling causes serious information loss in categories, containing a large number of images (a major category), leading to underfitting.

The re-weighting method focuses on training loss and is another important category rebalancing strategy. Re-weighting sets different weights for different categories of loss, setting larger weights for minor category loss, for example, and the weights can be adaptive (Huang et al., 2016; Wang et al., 2017). Among the many variants of this kind of method, the simplest is weighting according to the inverse of the number of categories (Huang et al., 2020); weighting according to the number of “effective” samples (Cui et al., 2019); and weighting according to the number of samples to optimize the classification interval (Cao et al., 2019). However, re-weighting is very sensitive to hyperparameters to a certain extent, which often leads to optimization difficulties, and re-weighting also has difficulties in handling large-scale real-world scenarios with imbalanced data (Mikolov et al., 2013).

In dealing with the problem of dataset imbalance, we can also learn from other learning strategies. With meta learning (domain adaptation), minor categories and major categories are processed differently to learn how to reweight adaptively (Shu et al., 2019), or to formulate domain adaptation problems (Jamal et al., 2020). Metric learning essentially models the boundary/margin near minor categories, with the aim of learning better embedding (Huang et al., 2016; Zhang et al., 2017). With transfer learning, major category samples and minor category samples are modeled separately, and the learned informativeness, representation, and knowledge of major category samples are transferred to minor category use (Liu et al., 2019; Yin et al., 2019). The data synthesis method generates “new” data similar to minor category samples (Chawla et al., 2002; Zhang et al., 2018). Decoupling features and classifier strategies can also be used. Recent studies have found out that feature learning and classifier learning can be decoupled, so that imbalanced learning can be divided into two stages. Normal sampling in the feature learning stage and balanced sampling in the classifier learning stage can bring better learning results (Kang et al., 2020; Zhou et al., 2020). This method of learning is the approach adopted in this work.

### Image Augmentation

Current random space image augmentation methods, such as image cropping and dropping, have a proven ability to improve effectively the accuracy of crop leaf disease classification. Recent studies have evaluated the image augmentation of image-based crop pest and disease classification, and explored the applicability of the image augmentation effect on specific datasets (Barbedo, 2019; Li et al., 2019). However, random image augmentation faces low efficiency and generates much uncontrolled noise, which may reduce training efficiency or affect feature extraction, such as dropping rice leaf regions, or cropping rice leaf backgrounds.

When using imbalanced datasets in the field of crop pests and diseases, some studies adopt simple image augmentation methods to augment images and balance datasets (Pandian et al., 2019; Kusrini et al., 2020), while other studies adopt GAN to generate related images and balance datasets (Douarre et al., 2019; Cap et al., 2020; Nazki et al., 2020; Zhu et al., 2020). Our image augmentation method focuses on spatially augmenting images of rice pests and diseases.

## Method

**Figure 2:**
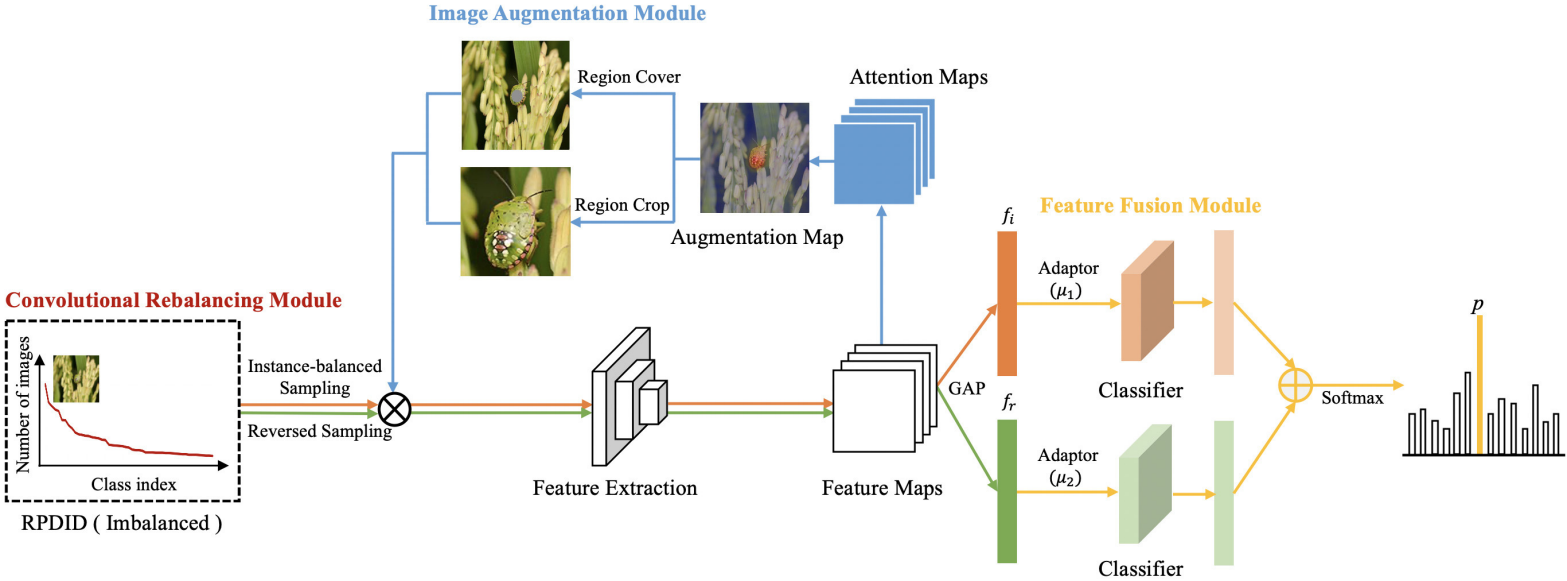
Overview of CRN.

In this section, we describe the proposed CRN in detail. First, to achieve feature learning and imbalance classification, we designed a CRM. The module proceeds as follows: Let *x* denotes the training sample and *y* the corresponding category label. Two sets of samples (*x*_*i*_, *y*_*i*_) and (*x*_*r*_, *y*_*r*_) are obtained by instance-balanced sampling and reversed sampling; these samples are then used as the input image of CRN. The corresponding feature maps are obtained after feature extraction, and attention maps are generated. At the same time, in order to augment images during training, we design an IAM. An attention map is chosen randomly to augment the image, including Region Cover and Region Crop. The samples of the two sampling methods and augmented images are used as input data for training. The feature maps undergo global average pooling (GAP) to obtain the corresponding feature vectors *f*_*i*_ and *f*_*r*_. Additionally, we design a FFM to fuse feature vectors. Finally, CRN uses SoftMax for predictive classification. The general structure of CRN is shown in Figure 2.

### Convolutional Rebalancing Module

We often encounter imbalanced datasets in our work on rice pest and disease classification. For this reason, we designed a CRM to improve classification performance.

#### Data Sampling

The CRM adopts instance-balanced sampling and reversed sampling to balance the impact of an imbalanced dataset. In instance-balanced sampling, each sample in the training set is only sampled once in an epoch with the same probability. Instance-balanced sampling retains the distribution characteristics of the data in the original dataset, so it is conducive to feature representation learning. Reversed sampling aims to alleviate the extreme imbalance between data samples and to improve the classification accuracy of minor categories. In reversed sampling, the sampling probability of each category is proportional to the inverse of the sample size; the smaller the sample size of a category, the greater the probability of being sampled.

We assume that there are a total of *D* categories in the dataset. The sample size of category *i* is *S*_*i*_, and the largest sample size in all categories is *S*_max_. For instance-balanced sampling, the probability *p*_*i*_ that each sample in the training set is sampled is as follows:

(1) $$\begin{array}{l} p_{i}=\frac{S_{i}}{\sum_{j=1}^{D}S_{j}} \end{array}$$

For reversed sampling, we first calculated the sampling probability $p_{i}^{\prime }$ of the *i*-th category according to the number of samples, as follows:

(2) $$\begin{array}{l} p_{i}^{\prime }=\frac{\frac{S_{\max}}{S_{i}}}{\sum_{j=1}^{D}\frac{S_{\max}}{S_{j}}} \end{array}$$

We then sampled randomly a category according to $p_{i}^{\prime }$, and finally took a sample from the *i*-th category to replace it. By repeating this reversed sampling process, we can obtain a mini-batch of training data.

#### Attention Representation

Here, we introduce the attention mechanism and increase the weight of the attention mechanism in the hidden layer of the neural network to accurately locate disease regions and the components of the pest object in the rice pest image (i.e., the spatial distribution of pest organs). Additionally, discriminative partial features are extracted to solve the classification problem. Our method first predicts partial regions where rice pests and diseases occur. Based on the attention mechanism, only image-level category annotations are used to predict the location of pests and diseases.

We use an advanced pre-trained CNN (EfficientNet-B0) as our backbone and choose the *MBConv6* (*stage6*) layer as feature maps. We denote *F* ∈ *R*^*H*×*W*×*C*^ as feature maps, where *H, W*, and *C* represent the height, width, and number of channels of the feature layer, respectively. Attention maps are obtained by 1 × 1 convolutional kernel. The attention maps *A* ∈ *R*^*H*×*W*×*M*^ obtained from *F* represent the location distribution of rice pests and diseases, as follows:

(3) $$\begin{array}{l} A=f\left(F\right)=\overset{M}{\underset{k=1}{⋃}}A_{k} \end{array}$$

In (3), *f*(·) is a convolution function, and ${\;A}_{K}\in R^{H\times W}$ represents a part of the rice pest or a visual graphic, such as the pest's head or another organ, and the diseased regions on the leaves. The number of attention maps is *M*.

We use attention maps instead of a region proposal network (Ren et al., 2017; Sun et al., 2018; Tang et al., 2018) to propose regions where pests and diseases occur in the image, because attention maps are flexible and can be more easily trained end-to-end in rice pest and disease classification tasks.

### Image Augmentation Module

Since the attention mechanism is used to better locate diseased regions and the position of the organ parts of the pest object in the image, the classification performance on images collected in the field is enhanced. At the same time, in order to further enhance performance, we design an IAM, which performs two kinds of processing: Region Crop and Region Cover. After the above processing, the raw image and augmented images will be trained as input data.

#### Augmentation Map

When there is a small number of regions where rice pests and diseases occur, the efficiency of random image augmentation is low, and a higher proportion of background noise is introduced. We use attention maps to augment the training data more effectively. Specifically, for each training image, we randomly select one of its attention maps *A*_*k*_ to guide image augmentation and normalize it as follows to the *k*-th augmentation map $A_{k}^{*}\in R^{H\times W}$, as follows:

(4) $$\begin{array}{l} A_{k}^{*}=\frac{A_{k}-mi\text{n}\left(A_{k}\right)}{\text{max}\left(A_{k}\right)-\text{min}\left(A_{k}\right)} \end{array}$$

#### Region Crop

Based on the augmentation map ${\;A}_{k}^{*}$, Region Crop randomly crops the discriminative region in the rice pest image and adjusts the size of the region to further extract its features. We obtain the cropping mask *C*_*k*_ from $A_{k}^{*}$. If $A_{k}^{*}\left(i,j\right)$ is greater than the threshold θ_*C*_ ∈ [0, 1], and then *C*_*k*_ is set to one; if less than or equal to the threshold, and then *C*_*k*_ is set to zero as in (5).

(5) $$\begin{array}{l} C_{k}\left(i,\;j\right)=1,\;if\;A_{k}^{*}\left(i,\;j\right)>\theta_{C} \end{array}$$

We then set a bounding box that can cover *C*_*k*_, and enlarge the region from the original image as the augmented input image. As the proportion of regions in the rice pest and disease images increases, it is possible to better extract more features from the regions where rice pests and diseases occur.

#### Region Cover

The attention regularization loss function, described below (Section Loss Function), supervises each attention map $A_{k}\in R^{H\times W}$ in representing the *k*-th region in the rice pest and disease images, but different attention maps may pay attention to regions where similar pests and diseases occur. To encourage attention maps to represent multiple occurrence regions of different pests and diseases, we propose Region Cover. Region Cover randomly covers a discriminative region in the rice pest and disease image, and then the image processed by the Region Cover operation is trained again. After that, when extracting features again, the features of other discriminative regions can be extracted, thereby prompting the model to extract more comprehensive feature. Specifically, in order to obtain the Region Cover mask $C_{k}^{\prime }$, we set $C_{k}^{\prime }$ to zero if $A_{k}^{*}\left(i,\;j\right)$ is greater than the threshold $\theta_{C^{\prime }}\in \left[0,\;1\right]$; otherwise, it is set to one.

(6) $$\begin{array}{l} C_{k}^{\prime }\left(i,\;j\right)=0,\;ifA_{k}^{*}\left(i,\;j\right)>\theta_{C^{\prime }} \end{array}$$

We use $C_{k}^{\prime }$ to cover the *k*-th region in the rice pest and disease images. Since the *k*-th region is covered, the IAM is required to propose other discriminative partial regions so that the robustness and location accuracy of the image classification can be improved.

### Feature Fusion Module

To fuse the features after GAP, we designed a novel FFM. The module controls the feature weight and classification loss *L* generated by the CRM and the IAM. The CRN first learns the features of the images in the RPDID according to the original distribution (instance-balanced sampling), and then gradually learns the features of the images in minor categories. Although, on the whole, feature representation, learning, and classifier learning should have the same importance, we believe that discriminative feature representation provides a basis for training a more robust classifier. Therefore, we introduce adaptive hyperparameters μ_1_ and μ_2_ into the training phase, where μ_1_ + μ_2_ = 1. We multiplied the image feature *f*_*i*_ extracted by instance-balanced sampling and image augmentation by μ_1_, and multiplied the image feature *f*_*r*_ extracted by inversed sampling and image augmentation by μ_2_. It should be noted that μ_1_ and μ_2_ are changed according to training epochs as in (7), where the current number of training epochs is defined as *E* and the total number of training epochs as *E*_*total*_.

(7) $$\mu_{1}=1-{(\frac{E}{E_{total}})}^{3}$$

As the number of training epochs increases, μ_1_ gradually decreases, causing CRN to gradually shift its focus from feature learning to classifier learning, which can exhaustively improve long-tailed classification accuracy; that is, from instance-balanced sampling to reversed sampling. Therefore, introducing the adaptive hyperparameters μ_1_ and μ_2_ into the entire training process enables CRN to fully focus on all categories of rice pests and diseases, and to further overcome the impact of an imbalanced dataset on the classification results.

### Testing Phase

In the testing process, rice pest and disease images with an unknown category are first sent to the CRM, and the feature vectors *f*_*i*_ and *f*_*r*_ are generated after GAP. We then set both μ_1_ and μ_2_ to 0.5 in FFM to balance the influence of different sampling methods on the prediction results. Additionally, features of equal weight are sent to their corresponding classifiers to obtain two predicted logits, and the two logits are aggregated by element-wise addition. Finally, the result is input into SoftMax to obtain the category of rice pests and diseases to which the image belongs.

### Loss Function

We define *x* as the training sample and *y* as the corresponding category label, where *y* ∈ {1, 2, ⋯ , *D*}, and *D* represents the total number of categories. First, we used the two sets of samples (*x*_*i*_, *y*_*i*_) and (*x*_*r*_, *y*_*r*_) obtained by instance-balanced sampling and reversed sampling as the input data of CRN. Then, after feature extraction, the corresponding feature maps were obtained and further attention maps were generated.

At the same time, the IAM augmented the image data during training. We randomly selected an attention map to augment the image, including Region Cover and Region Crop. Generally speaking, the samples were sampled in two ways, and the augmented data were used as input data for training. GAP was then performed on feature maps to obtain the corresponding feature vectors *f*_*i*_ and *f*_*r*_. Center loss has been proposed as a method of solving the problem of face recognition (Wen et al., 2016, 2019). Based on center loss, we designed a novel attention regularization loss function to supervise attention learning. We penalized variances of features belonging to partial regions of the same rice pest, which means that the partial features *f*_*i*_ and *f*_*r*_ can be close to the global feature center $c_{k}\in R^{1\times N}$, while attention map *A*_*k*_ can be activated at the same *k*-th partial region. The loss function of the IAM can be defined as follows:

(8) $$\begin{array}{l} L_{A}=\overset{M}{\underset{k=1}{\sum }}{\left\|(f_{i},\;f_{r})-c_{k}\right\|}_{2}^{2} \end{array}$$

In (8), *c*_*k*_ is the feature center of a partial region. We initialized *c*_*k*_ as zero and updated as follows:

(9) $$\begin{array}{l} c_{k+1}=c_{k}+\beta \left((f_{i},\;f_{r})-c_{k}\right) \end{array}$$

In (9), β adjusts the update rate of *c*_*k*_. The attention regularization loss function is merely applied to the original image.

As described above, the FFM fuses the features after GAP, where the adaptive hyperparameters are defined as μ_1_ and μ_2_. The weighted feature vectors μ_1_*f*_*i*_ and μ_2_*f*_*r*_ are sent to the corresponding classifiers $W_{i}\in R^{D\times C}$ and $W_{r}\in R^{D\times C}$, and the two outputs integrated together by element-wise addition. Therefore, the output logits *l* can be formulated as follows:

(10) $$\begin{array}{l} l=\mu_{1}{W_{i}^{\text{T}}f}_{i}+{\mu_{2}W_{r}^{\text{T}}f}_{r} \end{array}$$

CRN then uses SoftMax to calculate and output probability distribution as $p={\left[p_{1},p_{2},\ldots ,p_{D}\right]}^{\text{T}}$. We employed cross-entropy loss as classification loss:

(11) $$\begin{array}{l} L_{F}=-\overset{D}{\underset{y=1}{\sum }}\log(p_{y}) \end{array}$$

In summary, the loss function of CRN can be defined as (12), where λ is a hyperparameter (In our settings, λ = 1).

(12) $$\begin{array}{l} L_{CRN}={\lambda L}_{A}+{\mu_{1}L}_{F}(y_{i})+{\mu_{2}L}_{F}(y_{r}) \end{array}$$

The overall algorithm is summarized in Algorithm 1. We used the stochastic gradient method to optimize *L*_*CRN*_.

**Algorithm 1:**
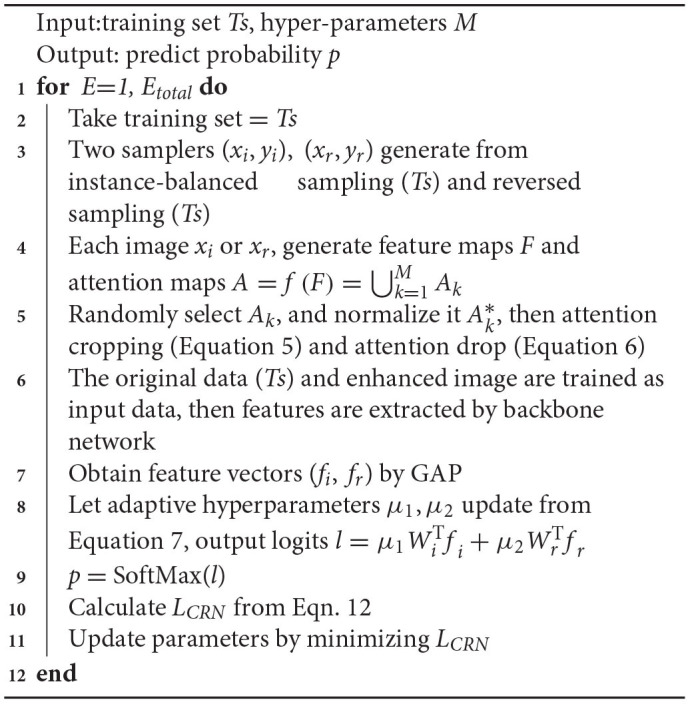
CRN algorithm.

## Experiments

### Datasets

**Figure 3:**
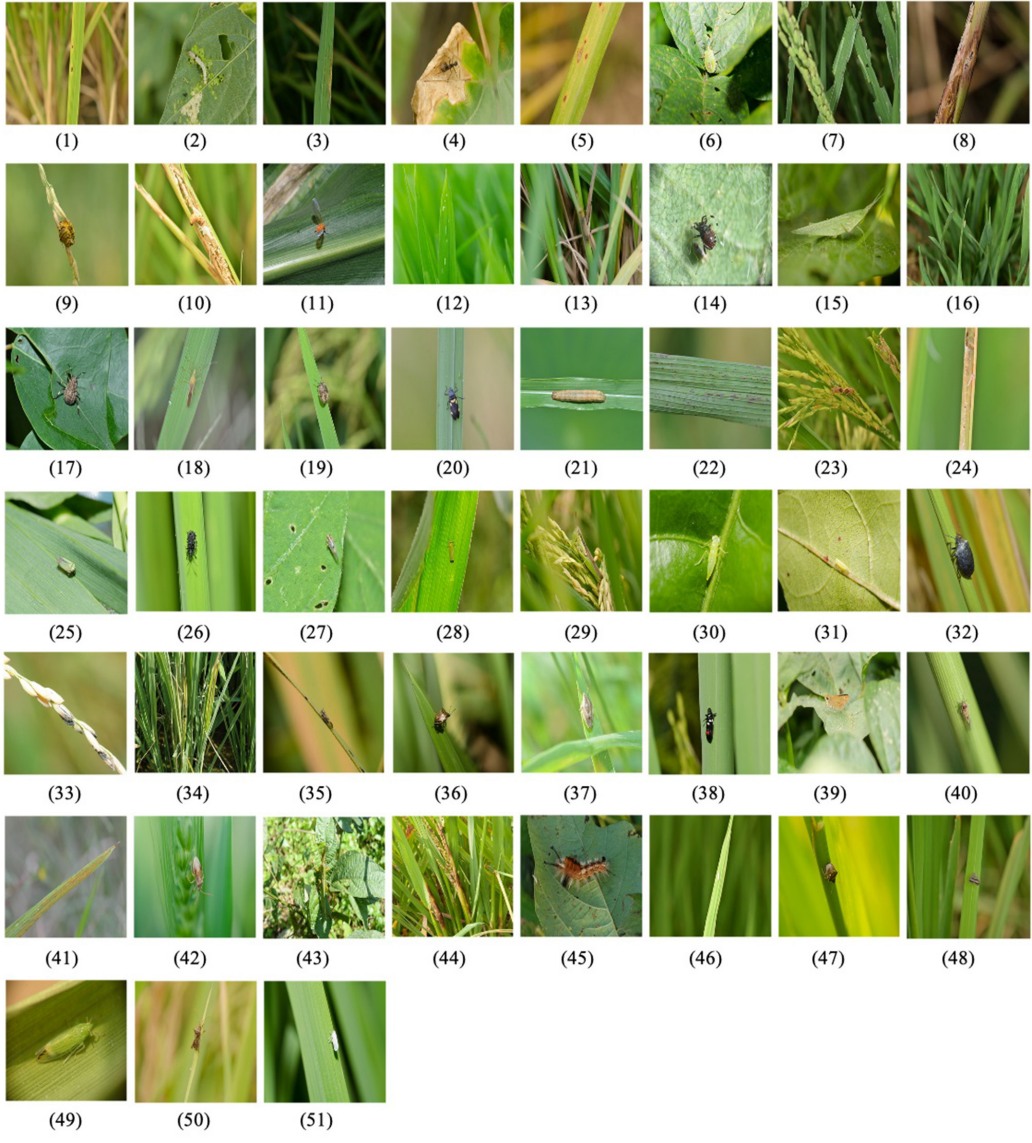
Examples of rice pests and diseases in RPDID. The number under each image corresponds to the category in Table 1, indicating the category to which the image belongs.

As China is the world's largest rice producer and consumer, the accurate classification of rice pests and diseases is particularly important for their prevention and control. To identify accurately the categories of rice pests and diseases in the field, we constructed the RPDID^1^ based on rice pests and disease images collected by the Institute of Agricultural Economy and Information, Anhui Academy of Agricultural Sciences, China. It contains 18,391 images of rice pests and diseases collected in the field and 51 categories, each with hundreds to thousands of high-quality images. Because the size of the original images is too large, we preprocess each RPDID image into a 512 × 512 size. Table 1 shows a statistical breakdown of the RPDID dataset. Figure 3 shows examples of rice pests and diseases in RPDID.

**Table 1:** RPDID dataset of rice pest and disease images collected in the field.

**Category**	**Type**	**Names of diseases and pests**	**Number of images**	**Category**	**Type**	**Names of diseases and pests**	**Number of images**
1	Disease	Rice blast	1,498	27	Pest	Laodelphax striatellus	206
2	Pest	Cotton leafworm	1,143	28	Pest	Black-tipped leafhopper	195
3	Disease	Bacterial leaf streak	1,060	29	Pest	Chinese rice grasshopper	195
4	Pest	Riptortus pedestris fabricius	999	30	Pest	Empoasca vitis gothe	182
5	Disease	Brown spot	971	31	Pest	Smaller green leafhopper	150
6	Pest	Green stink bug	792	32	Pest	Black rice bug	148
7	Pest	Rice leaf caterpillar	787	33	Disease	Rice kernel smut	147
8	Disease	Rice sclerotial stem rots	684	34	Disease	Stripe disease	145
9	Disease	Rice false smut	627	35	Pest	White backed planthopper	144
10	Pest	Pink rice borer	544	36	Pest	Eysarcoris montivagus dis.	144
11	Pest	Diostrombus politus uhler	509	37	Pest	Aelia nasuta wagner	139
12	Pest	Rice leaf roller	495	38	Pest	Rice spittle bug	137
13	Disease	Sheath blight	479	39	Pest	Parnara guttata brener	134
14	Pest	Chauliops fallax scott	473	40	Pest	Rice plant hoppers	125
15	Pest	Atractomorpha sinensis bolivar	453	41	Disease	Rice akagare	117
16	Pest	Psammotettix striatus l.	439	42	Pest	Rhopalus maculatus	114
17	Pest	Halyomorpha halys	426	43	Pest	Cotton grasshopper	112
18	Pest	Leptocorisa acuta	399	44	Disease	Sheath rot disease	105
19	Pest	Dolycoris baccarum	395	45	Pest	Cifuna locuples walker	105
20	Pest	Abidama liuensis metcalf	303	46	Pest	Recilia dorsalis	103
21	Pest	Oriental armyworm	292	47	Pest	Eysacoris guttiger	96
22	Disease	Leaf smut	283	48	Pest	Ricania taeniata cercopidae	92
23	Pest	Rice spiny coreid	280	49	Pest	Saccharosydne procerus matsumura	87
24	Pest	Chilo suppressalis	273	50	Pest	Clavigralla horrens dohrn	85
25	Pest	Rice leafhopper	255	51	Pest	Nisia afrovenosa meenoplidae	81
26	Pest	Rice hispa	244				
**Total**	**18,391**						

### Implementation Details

For comparison, our CRN uses EfficientNet-B0 as the backbone network for all experiments by standard mini-batch stochastic gradient descent with a momentum of 0.9 and a weight decay of 1 × 10^4^. For different pretrained networks, RPDID is preprocessed into the input sizes required by different networks (224 × 224; 299 × 299; 380 × 380). Except for the original division of the IP102 dataset, RPDID and other datasets are divided into a common distribution (80% for the training set and 20% for the test set). The attention maps are obtained through a 1 × 1 convolution kernel. We use GAP as the feature pooling function, and the thresholds θ_*C*_ and $\theta_{C^{\prime }}$ of Region Cover and Region Crop are both set to 0.5. We train all the models on multiple NVIDIA P100 GPUs for 500 epochs with a batch size of 32. The initial learning rate is set to 0.001, with exponential decay of 0.9 after every 10 epochs.

## Results

**Figure 4:**
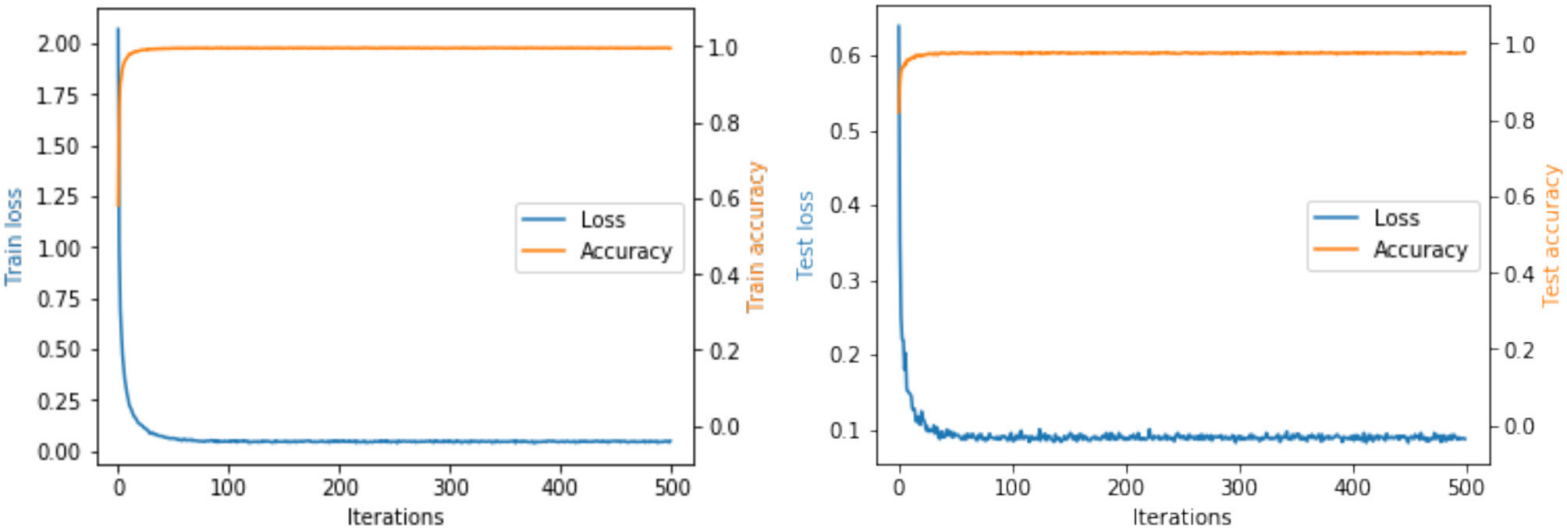
Accuracy and loss during CRN training and testing.

We have conducted extensive experiments on RPDID under imbalanced real-world scenarios. Figure 4 shows the accuracy and loss of our proposed CRN during training and testing. For the test set, when the number of epochs is 48, the loss converges to 0.09, and the accuracy is 97.58%. We find that CRN can achieve convergence and a higher level of accuracy in fewer epochs compared with state-of-the-art models, which proves that CRN has a strong ability to classify rice pest and disease images collected in the field.

### Comparison Methods

We fine-tune the pretrained ResNet-50, Inception-V3, EfficientNet-B0, and EfficientNet-B4 as benchmarks for comparison. Due to the lack of publicly available large-scale field crop pest and disease image datasets, we also compare our method with the latest methods on publicly available plant and pest image datasets. The results are shown in Table 2. It can be seen that our CRN has reached the latest level of accuracy on RPDID. In particular, compared with the backbone EfficientNet-B0, we have significantly improved the classification accuracy.

**Table 2:** Comparison with benchmarks and state-of-the-art methods on the test dataset.

**Methods**	**Accuracy (%)**
ResNet-50	81.31
Inception-V3	86.03
EfficientNet-B0	92.61
EfficientNet-B4	94.57
SpineNet-143 (Du et al., 2020)	95.82
FixSENet-154 (Touvron et al., 2019)	96.79
BiT-L (Kolesnikov et al., 2020)	97.16
EffNet-L2 (SAM) (Foret et al., 2021)	97.42
CRN	97.58

To further evaluate the performance of CRN, we conducted experiments on the publicly available plant image datasets Flavia (Wu et al., 2007), Swedish Leaf (Söderkvist, 2001) and UCI Leaf (Silva et al., 2013), and pest image dataset SMALL (Deng et al., 2018) and IP102 (Wu et al., 2019). Statistical information on the datasets is shown in Table 3. We used the training/test split described in section Implementation Details.

**Table 3:** Dataset statistics.

**Datasets**	**Categories**	**Training**	**Testing**
Flavia	32	1,526	381
Swedish Leaf	15	900	225
UCI Leaf	40	356	87
SMALL	10	450	113
IP102	102	52,603 (Train: 45,095 and Val: 7,508)	22,619

As Table 4 shows, our method outperforms current state-of-the-art methods on five datasets. Regardless of the dataset size, CRN can obtain a higher level of classification accuracy. Furthermore, it is proved that CRN has better performance across datasets.

**Table 4:** Accuracy of CRN on plant image datasets (Flavia, Swedish Leaf, and UCI Leaf) and pest image datasets (SMALL and IP102).

**Studies**	**Datasets**	**Accuracy (%)**	**Methods**
Murat et al. (2017)	Flavia	95.25	HOG, Moments, ANN, RF, SVM
	Swedish Leaf	99.89	
Saleem et al. (2019)	Flavia	99.48	AlexNet
Turkoglu and Hanbay (2019)	Flavia	98.94	Improved LBP
	Swedish Leaf	99.46	
Kaya et al. (2019)	Flavia	99.00	DF - VGG16/LDA
	Swedish	98.80	CNN - RNN
	UCI Leaf	96.20	DF - AlexNet/LDA
Nanni et al. (2020)	SMALL	92.43	Ensemble (AllSum)
	IP102	61.93	
Ayan et al. (2020)	SMALL	95.16	GAEnsemble
	IP102	67.13	
Our	Flavia	99.63	CRN
	Swedish Leaf	99.91	
	UCI Leaf	98.45	
	SMALL	97.36	
	IP102	70.42	

### Ablation Studies

#### Samplers for the CRM

To better understand CRN, we conducted experiments on different samplers used in the CRM. The classification accuracy of the models trained on RPDID with different samplers is shown in Table 5.

**Table 5:** Ablation study of different samplers used in CRM on RPDID.

**Samplers**	**Accuracy (%)**
Instance-balanced	95.13
Class-balanced	95.84
Reversed	94.65
CRN method	97.58

We used the following samplers. (1) Instance-balanced sampling, where every training sample has an equal chance of being selected. (2) Class-balanced sampling, where each category has the same probability of being selected. Each category is selected fairly, and samples are selected from the category to construct mini-batch training data. (3) Reversed sampling, where the sampling probability of each category is inversely proportional to the sample size. The smaller the sample size of a certain category, the more likely it is to be sampled. (4) Our CRM combines instance-balanced sampling and reversed sampling.

We can find from Table 5 that when a better sampling strategy is used, the performance can be better. The sampling method we use can provide better results than single instance-balanced sampling. We believe that instance-balanced sampling provides general feature representation. With adaptive hyperparameter μ_1_ decreasing, the main emphasis of the CMR in CRN turns from the feature learning to the classifier learning (from instance-balanced sampling to reversed sampling), then the reversed sampling can be more concerned with minor categories. Our results for different sampling strategies on training validate our works that try to design a better image sampling method.

#### Accuracy Contribution

The proposed CRN is composed of three modules: CRM, IAM, and FFM. To study the contribution of the three modules to classification accuracy, we conducted related experiments on RPDID. We fine-tune the pretrained EfficientNet-B0 and use cross entropy (CE) for training to use it as a baseline. Accordingly, we add and adjust other modules for comparison. As shown in Table 6, the results prove that all three modules of our CRN can improve effectively the classification accuracy of rice pests and disease images, and that the attention-guided IAM is more effective than random image augmentation (RIA).

**Table 6:** Contribution of proposed components and their combinations.

**Modules**	**Accuracy(%)**
EfficientNet-B0 and CE	94.57
CRM and FFM	96.04
CRM and RIA and FFM	96.43
CRM and IAM and FFM	97.58

#### Effect of Number of Attention Maps

Discriminative regions usually help to represent the object; hence, a larger number of discriminative regions can help to improve the classification performance (Wang et al., 2019; Yang et al., 2020a). We use different numbers of attention maps (*M*) for experiments, as shown in Table 7. It can be seen that as *M* increases, the classification accuracy also increases. When *M* reaches 32, the classification accuracy rate reaches 97.72%. However, if *M* continues to increase, the increase in classification accuracy is limited and the feature dimensionality of a discriminative region almost doubles. IAM in CRN can set the number of discriminative partial regions in rice pest and disease images, and increase *M* within a certain range to obtain more accurate classification results.

**Table 7:** Classification accuracy of different numbers of attention maps on RPDID.

***M***	**Accuracy(%)**
4	96.17
8	96.93
16	97.58
32	97.72

#### Visualization of the Effect of IAM

**Figure 5:**
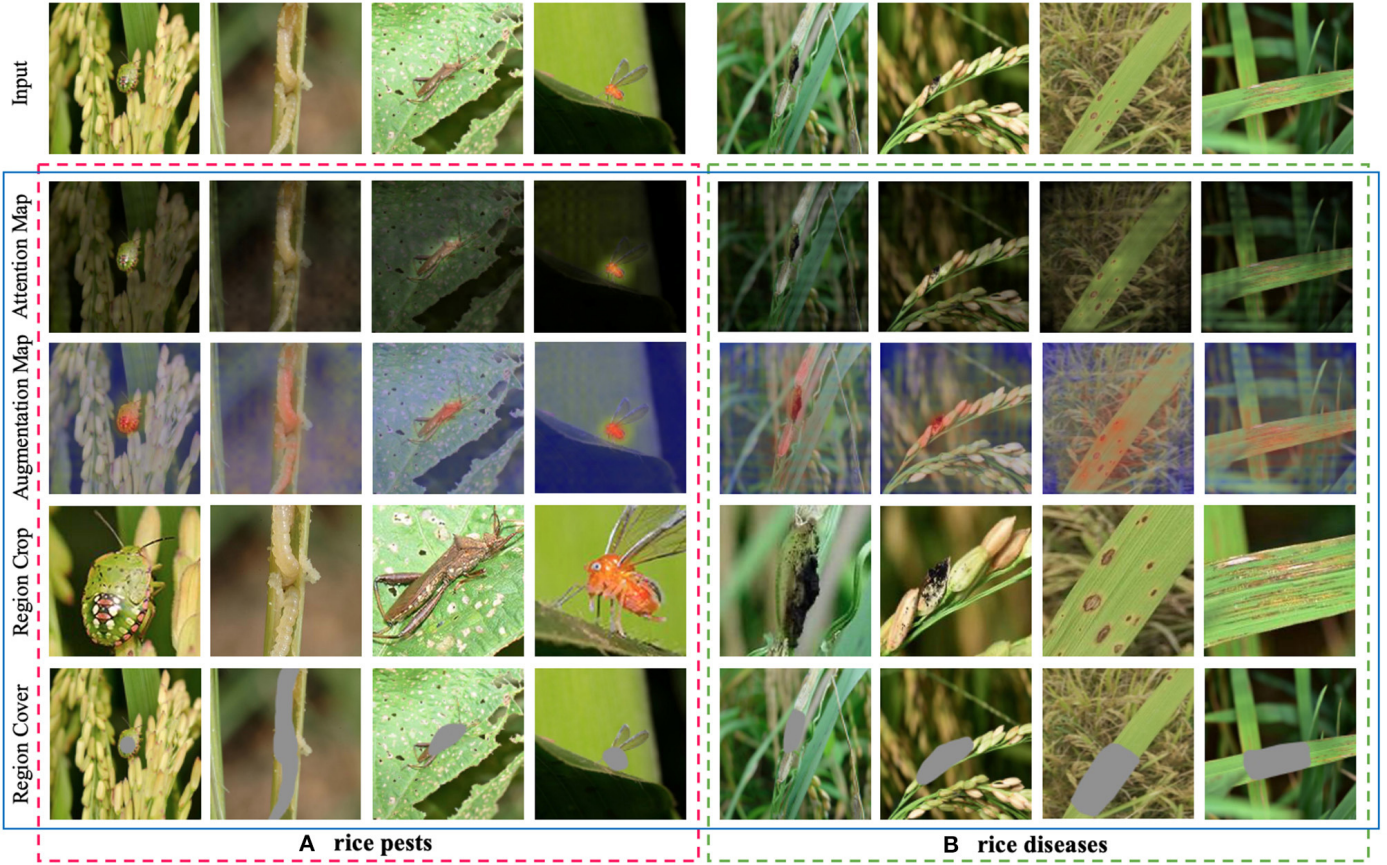
Visualization of the effect of image augmentation in CRN on rice pest and disease images. **(A)** rice pests. **(B)** rice diseases.

To analyze the image augmentation effect of IAM in CRN, we draw discriminative regions predicted by IAM through Region Cover and Region Crop. In Figure 5, we perform image augmentation on rice pest and disease images. All images in the first row are original images; all images in the second row are attention maps; the images in the third row are augmentation maps after attention learning; and the images in the fourth and fifth rows are images after image augmentation operations (Region Crop and Region Cover).

We can see that where pests and diseases occur in certain regions; these discriminative regions are highlighted in augmentation maps. From the fourth row in Figure 5A, we can clearly see that the discriminative region in the image after Region Crop is enlarged. From the fifth row in Figure 5A, the discriminative regions of the pest are the head and body parts, which is consistent with human perception. From the fourth row in Figure 5B, we can see that, although it is quite difficult to identify rice disease regions in the field, IAM can still find discriminative regions from the image. From the fifth row in Figure 5B, we can see that IAM can accurately cover some discriminative regions, thereby prompting CRN to find more discriminative regions, which is especially helpful to the classification effect.

## Conclusion

This paper has proposed a CRN in order to study the classification of rice pest and disease images in imbalanced datasets. The results show that the combination of the CRM, IAM, and FFM enhances the classification of rice pests and disease images collected in the field. Extensive experiments on common plant datasets and RPDID for imbalanced classification have demonstrated that CRN outperforms state-of-the-art methods. CRN can be further applied in production practice to provide support for the intelligent control of rice pests and diseases.

## Data Availability Statement

The data analyzed in this study is subject to the following licenses/restrictions: Our data is protected by copyright. For data sources, please contact the Institute of Agricultural Economy and Information, Anhui Academy of Agricultural Sciences' website at: http://jxs.aaas.org.cn/.

## Author Contributions

GY and GC: conceptualization, methodology, software, formal analysis, data curation, writing, original draft preparation, and visualization. GY, GC, and CL: validation. GY and YG: investigation. GC, CL, and JF: resources, supervision, and project administration. GY and HL: writing, review, and editing. All the authors contributed to the article and approved the submitted version.

## Conflict of Interest

The authors declare that the research was conducted in the absence of any commercial or financial relationships that could be construed as a potential conflict of interest.

## Footnotes

^1^RPDID is non-public. For data sources, please contact the Institute of Agricultural Economy and Information, Anhui Academy of Agricultural Sciences' website at: http://jxs.aaas.org.cn/.
